# Highly Efficient and Recyclable Catalysts for Cellobiose Hydrolysis: Systematic Comparison of Carbon Nanomaterials Functionalized With Benzyl Sulfonic Acids

**DOI:** 10.3389/fchem.2020.00347

**Published:** 2020-04-27

**Authors:** Samuel Carlier, Sophie Hermans

**Affiliations:** Université Catholique de Louvain, IMCN Institute, Louvain-la-Neuve, Belgium

**Keywords:** biomass conversion, cellulose, cellobiose, glucose, hydrolysis, carbon, sulfonic acid, graphene

## Abstract

Carbon materials such as activated coal, nanotubes, nanofibers, or graphene nanoplatelets were functionalized with sulfonic acid moieties by a diazonium coupling strategy. High acidity was obtained for the majority of the carbon solids except for the carbon nanofibers. The activity of these acidic catalysts for the hydrolysis of cellobiose, as model molecule for cellulose, into glucose in neutral water medium was studied. The conversion of cellobiose is increasing with the acidity of the catalyst. We found that a minimum threshold amount of acidic functions is required for triggering the hydrolysis. The selectivity toward glucose is very high as soon as sulfonic functions are present on the catalyst. The robustness of the sulfonic functions grafted on the carbons has been highlighted by successful recyclability over six runs.

## Introduction

**Figure 1:**
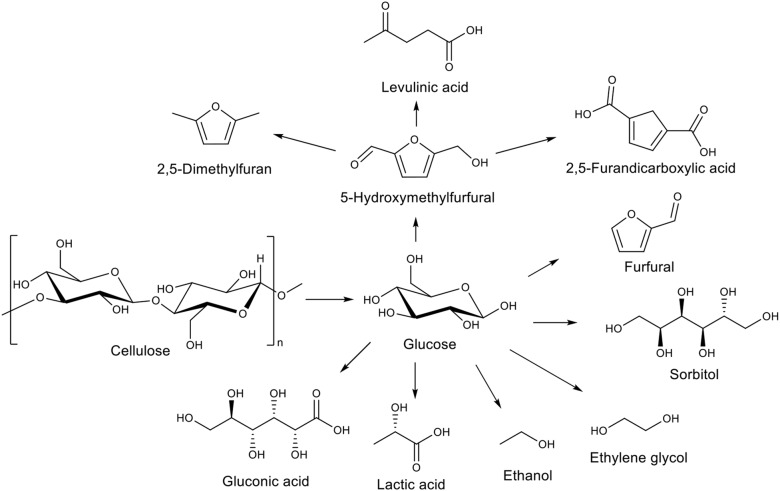
Glucose as a chemical platform.

The increasing demography and concomitant energy demand requires finding sustainable production routes for fuels and chemicals. Nowadays the lignocellulosic biomass is the most abundant renewable resource and the perfect candidate to solve this challenge, as it is not competing with food. The lignocellulosic biomass originates from forest or agricultural wastes and is composed of three polymers: lignin (10–30%), hemi-cellulose (15–40%), and cellulose (30–65%) (Chaturvedi and Verma, 2013; Sarra et al., 2016). Lignin is a randomly-linked phenylpropanoid polymer. Hemi-cellulose is a non-linear polymer of pentoses, hexoses and sugar acids. Cellulose is a linear polymer only composed of glucose units. These three polymers can lead to many interesting products such as furfural or xylitol (from hemicellulose; Menon et al., 2010), glucose, 5-hydroxymethylfurfural or sorbitol (from cellulose; Ruppert et al., 2012), aromatic alcohols, or aromatic aldehydes (from lignin; Li et al., 2016; Wang et al., 2019). In particular, the main reason behind the high interest for cellulose hydrolysis into glucose is that glucose is a chemical platform. It can be further transformed into various added-value chemicals (Kobayashi et al., 2011), as depicted in Figure 1. Depending on the metal choice, catalysts for these further transformations can be tuned to obtain the desired product. For example, sorbitol is obtained by glucose hydrogenation using Ru (Lazaridis et al., 2015) or gluconic acid is produced by glucose oxidation using Au (Biella et al., 2002; Hermans et al., 2011) or Pd (Haynes et al., 2017).

Cellulose is a polymer of glucose connected by β-1,4-glycosidic bonds, which presents a very high molecular weight (from 300,000 to 500,000 g/mol). Cellulose has a microcrystalline structure with numerous hydrogen bonds between molecules (intra and inter-molecules but also inter-sheets) that makes it insoluble in most solvents (Dhepe and Fukuoka, 2008). For these reasons, depolymerization of cellulose remains a tricky challenge. Industrially, the most common way of processing cellulose is by using homogeneous mineral acids such as HCl or H_2_SO_4_. The drawbacks of this method are the corrosion of the reaction vessels but also waste production and disposal (Gitifar et al., 2013). Enzymes, such as cellulases, are very effective for the hydrolysis of cellulose. Indeed they are faster, more selective, can be used at room temperature and do not generate waste (Philippidis et al., 1993). Unfortunately, their recyclability is poor and they do not sustain high temperatures, making it difficult to use them industrially (Guarín et al., 2018). Despite the fact that they are less active and less specific than the homogeneous ones, heterogeneous catalysts are being studied to overcome the recovery and stability issues. Many catalytic solid materials have been investigated in this context, such as metal oxides (Huang and Fu, 2013), Amberlyst-15 resin (Suganuma et al., 2010), zeolites (Huang and Fu, 2013), hydrotalcites (Guarín et al., 2018), functionalized carbon materials (Onda et al., 2009), etc. However, heterogeneous catalysts in general still suffer from deactivation problems like poisoning or leaching of the active sites (Argyle and Bartholomew, 2015).

**Figure 2:**
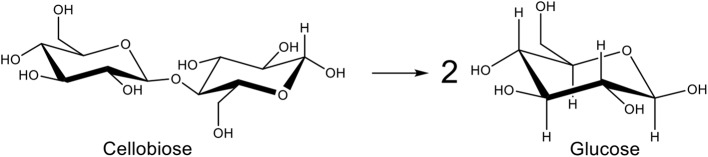
Hydrolysis of cellobiose into glucose.

These catalysts can be assisted by other ways of breaking the cellulose backbone. Supercritical water display physical properties that are different from liquid water and can be used to dissolve cellulose (Tolonen et al., 2015). Another way of dissolving cellulose is the use of ionic liquids. These show interesting properties such as low vapor pressure, high thermal stability, and high solvation ability. They efficiently dissolve cellulose because of the formation of bonds between the hydroxyl group H atoms in the biopolymer and the anions of ionic liquids. This leads to the breaking of the intermolecular bonds in cellulose (Novoselov et al., 2007). However ionic liquids are very costly, toxic and corrosive (Wang et al., 2012). Crystallinity of cellulose can be decreased by ball-milling, leading to amorphization and easier hydrolysis (Yu and Wu, 2011). Microwave radiations is also a very popular technique. When they hit cellulose, microwaves create hot spots that reduce the crystallinity of cellulose and increase its degradation under mild conditions (Liu et al., 2018). For all these reasons, cellobiose (only two glucose units held together by β-1,4-glycosidic bond), which is soluble in water, is chosen as a model-molecule of cellulose in many studies (Figure 2) (Deng et al., 2012).

Herein, we report an efficient process for the selective hydrolysis of cellobiose into glucose (Figure 2) using acidic (nano)carbon materials. Depending on the production method, carbon materials already possess acidic functions such as phenols, carboxylic acids, or lactones. However, these acidic functions are not strong enough or not present in sufficient amount to catalyze the hydrolysis of the glycosidic bond. Strong acid sites like sulfonic acids are needed. Usually, carbon solids functionalized by H_2_SO_4_ will give labile sulfonic functions that are susceptible to be leached away during catalytic tests. Moreover, the structural integrity of the treated carbon is usually weakened. Various methods to functionalize carbon solids exist (Yang et al., 2019) but in this work the functionalization strategy we chose to introduce surface sulfonic functions is leading to a strong covalent C-C bond with the carbon surface, without damaging its backbone structure. It involves diazonium coupling reaction with an organic starting compound rather than the corrosive inorganic sulfuric acid or a mixture of nitric/sulfuric that is commonly used also. This functionalization strategy has been tested on CNF, CNT, CB, and activated or mesoporous carbons in separated reports (Price and Tour, 2006; Toupin and Bélanger, 2007; Wang et al., 2007; Liu et al., 2010; Stellwagen et al., 2013) but never applied to this particular reaction. Therefore, the aim of this work is to show that the functionalization can be applied to almost all types of carbon (nano)materials in a systematic and comparative manner, and used for a biomass valorization reaction. We will show the high activity and recyclability of the obtained materials for cellobiose hydrolysis.

## Materials and Methods

### Reagents and Materials

The activated coal (SX+) was obtained from NORIT. The reduced graphene oxide N002-PDR (RGO) was obtained from Angstron Materials. The carbon multi-walled nanotubes (MWCNT) were obtained from Nanocyl (Belgium) (NC 7000 Thin MWCNTs, 95+% C purity). The carbon black (CB) was obtained as 250 G type from IMERYS GRAPHITE & CARBON. The carbon nanofibers (CNF; LHT type) were obtained from Applied Sciences Inc (USA). The graphene nanoplatelets (GNP; M15, and C750 types) were purchased from Sigma-Aldrich. Sulfanilic acid (99%), isopentyl nitrite (96%), D-(+)-cellobiose (≥99%), cellulose (reference C6288, crystalline and high purity) were also supplied by Sigma-Aldrich and used as received.

### Catalyst Preparation

The materials functionalization was carried out by a diazonium coupling method (Stellwagen et al., 2013). Typically, 1 g of carbon solid was dispersed in 60 mL distilled water. 1.5 g of sulfanilic acid were added and the suspension was stirred at 70°C during 10 min. 1.2 mL of isopentyl nitrite were added at 30°C and the mixture was stirred during 16 h. Then it was filtered out and washed with distilled water and ethanol. The resulting material was dried overnight at 100°C.

All the functionalized support samples will be specified by the acronym SO_3_H followed by the acronym of the support. For example, functionalized activated carbon SX+ will be named SO_3_H/SX.

### Characterization Methods

XPS analyses were carried out at room temperature with a SSI-Xprobe (SSX 100/206) photoelectron spectrometer from Surface Science Instruments (USA), equipped with a monochromatized microfocus Al Xray source. Samples were stuck onto small sample holders with double face adhesive tape and then placed on an insulating ceramic carousel (Macor®, Switzerland). Charge effects were avoided by placing a nickel grid above the samples and using a flood gun set at 8 eV. The binding energies were calculated with respect to the C-(C, H) component of the C1s peak fixed at 284.4 eV. Data treatment was performed using the CasaXPS program (Casa Software Ltd., UK). The peaks were decomposed into a sum of Gaussian/Lorentzian (85/15) after subtraction of a Shirley-type baseline.

TPD-NH_3_ analyses were performed on Hiden Catlab reactor combined with a QGA Hiden quadrupole mass spectrometer. Samples were pretreated under Ar (30 ml/min, 5.0 AirLiquide) at 200°C during 2 h to remove all the water before the analysis. NH_3_ adsorption was performed at 70°C during 1 h by flowing a mixture of Argon (20 ml/min) and 5% NH_3_ in He (10 ml/min). The catalyst was then flushed in Ar (30 ml/min) during 2 h and then, the NH_3_ desorption measurement was performed under Ar (30 ml/min) from 70 to 600°C (heating rate of 10°C /min).

Boehm titration method was used to evaluate the catalysts acidity (Boehm et al., 1964; Goertzen et al., 2010). NaOH solutions were prepared by dilution of Titrisol ampoules (VWR) containing precise and known quantities of sodium hydroxide. HCl solutions were prepared by the dilution of concentrated hydrochloric acid. The HCl concentrations were determined by titration with the standard NaOH solutions. These solutions were prepared with mQ water that had been previously decarbonated by nitrogen flushing. For titrating the acid groups, 60 mg of sample were dispersed in 30 mL of NaOH 0.01 mol/L and the solution was decarbonized for 1 h under Ar flux. The mixture was then agitated for 23 h under Ar atmosphere. The suspension was then filtrated and two times 10 mL of the resulting filtrate were back-titrated, under Ar flux, using the HCl 0.005 mol/L solution. The indicator used is phenolphthalein. The amount of acid functions on the catalyst is determined by calculating the difference between the initial amount of NaOH and the amount of NaOH titrated by the HCl.

Total organic content (TOC) analyses have been performed on each solution after catalytic test by using a Shimadzu TOC-L analyzer with a ASI-L autosampler using the combustion catalytic oxidation method.

### Catalytic Tests

The tests were carried out in a 250 mL stainless steel Parr autoclave. 1 g of cellobiose was added to 300 mg of catalyst in 120 mL of mQ water. Indeed cellobiose is soluble in water and we also need water as a reactant for the hydrolysis. Then, the autoclave was sealed and the system was purged three times with nitrogen and heated up to 403 K under autogenic nitrogen pressure (6–7 bar). At that controlled temperature, the agitation was started at 1,700 rpm for 2 h. The system was then cooled down to room temperature and the solution was filtrated. The filtrate was then diluted to 250 mL with mQ water and analyzed by HPLC. The catalytic testing conditions, adapted from literature (Delidovich and Palkovits, 2016), were chosen to avoid reaching 100% of conversion during the reaction duration, in order to be able to compare the different catalysts. It is known that, ideally, selectivities have to be compared at the same conversion rate but we are comparing here all our results after the same reaction time (2 h), which is pertinent as well, especially when considering productivity. For the recyclability tests, the filtrated catalyst was dried in an oven at 373 K overnight and it was re-used directly using the same testing conditions.

HPLC analyses were performed with a Waters system equipped with Waters 2414 refractive index (RI) detector (detector temperature = 30°C). Two columns were used. The first one is a Carbohydrate Transgenomic CarboSep CHO682 column, with mQ H2O (18 MΩ.cm at 25°C) as eluent, a flux of 0.4 mL/min, a column temperature of 80°C and 20 μL of injected volume. The second one is an Aminex HPX 87C column, with mQ H2O (18 MΩ.cm at 25°C) as eluent, a flux of 0.5 mL/min, a column temperature of 85°C and 25 μL of injected volume.

The conversion of cellobiose was calculated as follows:

$$\begin{array}{l} Cellobiose\;conversion\;\left(\%\right)=\;\frac{n\;cellobiose\;converted}{n\;cellobiose\;engaged\;}*100 \end{array}$$

The selectivity in glucose was calculated as follows:

$$\begin{array}{l} Selectivity\;in\;glucose\;\left(\%\right)=\;\frac{n\;glucose\;producted}{2*\left(n\;cellobiose\;converted\right)}*100\; \end{array}$$

## Results and Discussion

### Functionalization

**Figure 3:**
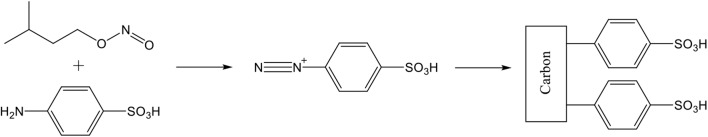
Diazonium coupling on carbon materials for sulfonic acid grafting.

Several carbon materials have been functionalized by a diazonium coupling strategy. First sulfanilic acid and isoamyl nitrite react to form *in situ* a diazonium cation. After that step, a de-diazoniation reaction will couple the aryl sulfonic acid fragment on the carbon surface, *via* a strong covalent C-C bond with C atoms in the polyaromatic structure of the carbon material backbone (Figure 3). This functionalization method is safer than common methods (involving sulfuric acid) for producing sulfonic functions on carbon materials and can easily be scaled up. Moreover, this method is gentle on the carbonaceous (nano-)material that keeps its mechanical strength.

**Figure 4:**
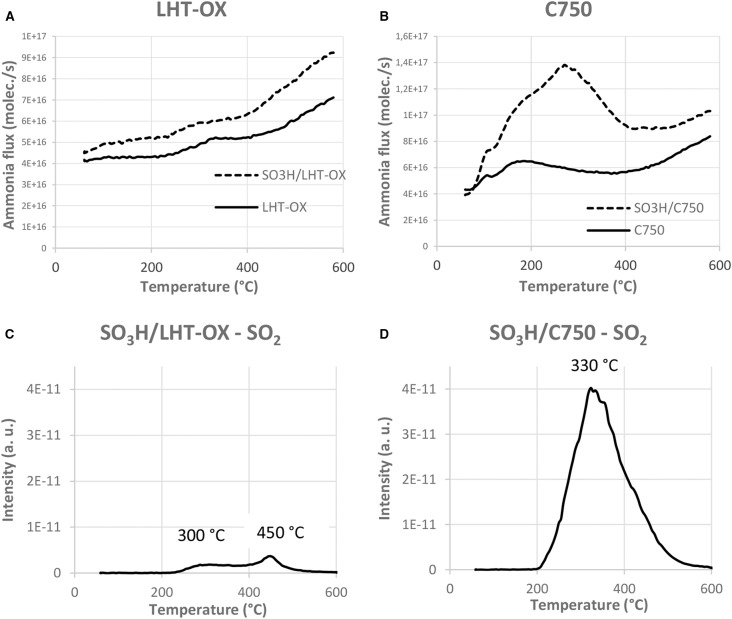
Ammonia desorption flux as a function of the temperature **(A,B)** and SO_2_ flux followed by MS during the TPD analysis **(C,D)**.

**Figure 5:**
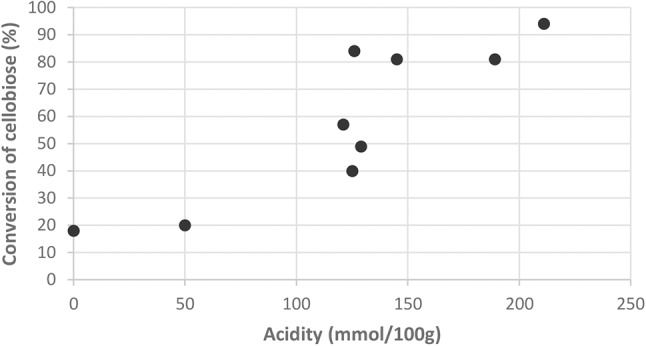
Conversion of cellobiose depending on the acidity of the catalyst.

**Figure 6:**
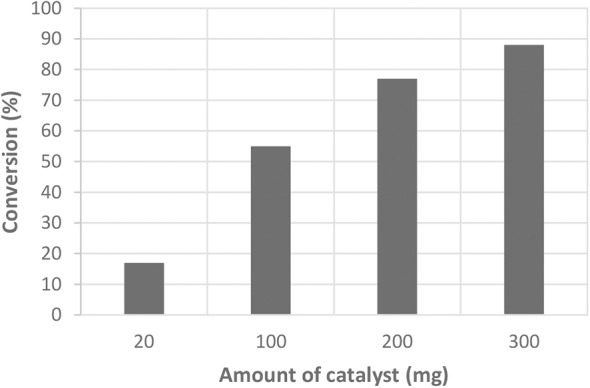
Conversion of cellobiose depending on the amount of catalyst (SO_3_H/SX+).

**Figure 7:**
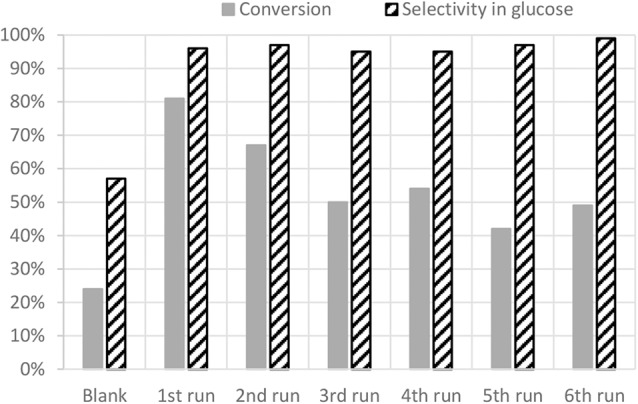
Recyclability of SO_3_H/SX+ catalyst.

A first observation is that the functionalized carbons become more hydrophilic and give better suspensions in aqueous medium. Functionalized materials have been characterized by XPS and the spectra displayed a single S_2p_ peak, at 168.3 eV, which can be assigned to sulfonic functions (Wu et al., 2010). All the selected carbon materials have been successfully functionalized and the XPS spectra of each sample, before and after functionalization, are presented in the Electronic (Supplementary Information S1, Figures 1–**7**). Most carbon materials do not display any S_2p_ peak before functionalization except GNP M15 that has a S_2p_ peak around 164 eV which could be identified as thiol functions (Castner et al., 1996). These thiols functions might arise during synthesis of GNP M15. After functionalization, all carbon materials present a huge peak at 168.3 eV corresponding to sulfonic functions, as expected. The obtained S/C and N/C surface atomic ratios are presented in Table 1. It is observed that the S/C ratio is increasing strongly after functionalization, confirming the grafting of sulfonic functions. In addition, the N/C ratio is also rising after the reaction. This is due to some isoamyl nitrite that would have not reacted completely during the functionalization procedure and is still adsorbed on the carbon surface. This is confirmed by XPS analyses. Indeed, a nitrogen peak is appearing after the functionalization but it is not present anymore after catalytic test (Supplementary Information S2). Moreover, the XPS results have shown that the carbon peak has not been altered (Supplementary Information S2). The integrity of the carbon support is intact after functionalization.

**Table 1:** Catalysts characterization data: specific surface area; acidity determined via Boehm titration; atomic ratios determined by XPS analyses.

**Sample**	**Specific area (S_**BET**_, m^2^/g)**	**Acidity (mmol/100 g)**	**S/C (%at. ratio)**	**N/C (%at. ratio)**
LHT-OX	26	10	0.001	0.002
SO_3_H/LHT-OX		50	0.019	0.015
RGO	600	73	0.002	0
SO_3_H/RGO		126	0.021	0.004
SX+	922	42	0	0.002
SO_3_H/SX+		145	0.042	0.058
MWCNT	257	14	0	0
SO_3_H/MWCNT		189	0.018	0.004
CB	63	74	0	0.004
SO_3_H/CB		121	0.025	0.041
GNP-M15	116	136	0.005	0
SO_3_H/GNP-M15		125	0.014	0.024
GNP-C750	799	228	0	0
SO_3_H/GNP-C750		211	0.030	0.044

The acidity of each catalyst was determined by Boehm titration (Table 1). For each different carbon, the acidity after functionalization is more important than before. The non-functionalized materials are presenting a low acidity, except the GNPs that already possess acidic functions (XG Sciences Inc., 2018). Indeed, graphene nanoplatelets (GNPs) are produced by graphite exfoliation in acidic medium and are therefore heavily functionalized. The increase is more or less important depending on the type of carbon considered. Indeed the diazonium coupling reaction is grafting the sulfonic functions directly on the carbon aromatic rings. The amount of defects on the carbon should not influence the yield of this grafting reaction.

There is one exception: the GNPs that keep almost the same acidity after the sulfonic functions grafting. This can be explained by the huge amount of acidic groups already present on the surface at the start. This does not mean that the functionalization is not occurring. Indeed, some of the starting acidic functions have been replaced by the sulfonic functions and this is confirmed by the XPS results showing an increase in the sulfur amount. Therefore, the acidity displayed by the GNPs is not coming only from the sulfonic functions but also from weaker O-only acidic functions.

The grafting disparities between some of the carbon solids may be linked to their specific surface areas, especially the CNF (LHT-OX) that displays a low surface area (26 m^2^/g). By opposition, activated coal SX+, which has a high specific surface area (922 m^2^/g), is showing a much higher acidity increase. Nevertheless, a perfectly linear relationship between BET surface area and the obtained acidity was not observed (Supplementary Information S3). Indeed, there are other factors that can impact the effectiveness of the functionalization. The type of carbon surface is important, i.e., the amount of defects, the presence of an amorphous layer, the number of Csp2 carbon atoms etc., but also the amount of acid functions already present at the beginning. Moreover, the diazonium produced *in situ* might have some difficulties to access the internal surface due to microporosity in some materials.

If we look at other catalysts synthesized by the same method in the literature, we can see that the acidity reported for nanofibers [52 or 63 mmol/100 g; (Stellwagen et al., 2013) is similar to our SO_3_H/LHT-OX catalyst, which is the lowest value obtained here after functionalization. Moreover our best catalysts, especially the functionalized MWCNT that reaches 189 mmol/100 g starting from a mere 14 mmol/100 g acidity, possess higher acidity than reported sulfonated carbons (108 mmol/100 g) or acidic aluminosilicates (105 mmol/100 g) but not as much of course as Amberlyst-15 [470 mmol/100 g; (Onda et al., 2008; Huang and Fu, 2013; Stellwagen et al., 2013). However, the sulfonic functions on our catalysts are more accessible and should display a better hydrolysis activity. A more detailed Table compiling acidic catalysts from the literature and their acidity values can be found in the Supplementary Information (S4).

TPD analyses have been performed to assess the strength of the acidic sites. Data are all presented in the Supplementary Information (S5). We can observe in the representative examples of Figure 4 that the functionalized LHT-OX presents the same curve than without functionalization (Figure 4A). It confirms the very low acidity of the functionalized material due to poor grafting of the sulfonic sites. We can also determine the presence of two types of acidic functions on the functionalized C750 (Figure 4B). Indeed a peak around 180°C (weak acids) can be noticed for the starting C750. After functionalization this peak is still visible but a more intense peak appears at 300°C. The first peak corresponds to the weak acidic functions already present on the carbon surface while the second peak can be identified as the sulfonic functions and can also be found on all the other functionalized materials. This peak from the sulfonic functions confirms the hypothesis that weak acids are replaced by the sulfonic acids in the case of M15 and C750 GNPs.

However, TPD analyses do not seem to be ideal for sulfonic acids characterization because of the sulfonic functions thermal degradation, as mentioned in the literature (Shimizu et al., 2005). Indeed the SO_2_ peak has been followed by MS during the TPD analyses and for each functionalized material a peak at 300°C is observed (Figures 4C,D and Supplementary Information S6). This indicates that the sulfonic functions are degraded at this temperature. This means that the ammonia measured at 300°C comes from the sulfonic functions degradation, rather than desorption from intact sites. If the sulfonic functions were stable at higher temperature, the ammonia desorption could possibly occur at higher temperature. Therefore, we certainly underestimate the strength of these functions if concluding solely on the basis of the peak observed in TPD analyses and corresponding temperature. We cannot reliably characterize the sulfonic functions with this method, and find Boehm titrations more pertinent.

### Catalysis

The catalysts have been tested for the hydrolysis of cellobiose into glucose (Figure 2) under mild conditions, namely under N_2_ autogenic pressure, at 130°C and 1,700 rpm for 2 h (Table 2). Blank was carried out using the same testing conditions, as described in the experimental part, but without any catalyst. Globally, non-functionalized starting carbon materials are giving the same conversion than the blank, around 25%. It confirms that the acidity determined by Boehm titration is due to weak acids, even with GNPs, that cannot catalyze the hydrolysis reaction. Indeed the observed conversion arises from the thermal cleavage of the glycosidic bond at this temperature. Functionalized catalysts are dramatically increasing the conversion up to a maximum of 94% with the SO_3_H/GNP-C750 graphene material. Sulfonic functions are controlling the hydrolysis pathway [(Huber et al., 2006); the mechanism is shown in Supplementary Information S7 and are improving the selectivity toward glucose to 95% (vs. 57% in the “blank” case). It is noteworthy that these figures (>90% conversion and selectivity) were obtained in only 2 h at 130°C. Common side-products usually obtained from glucose such as levulinic acid or formic acid have not been observed here. Other products such as 5-hydroxymethylfurfural cannot be completely ruled out as they are not identified with the HPLC column used in this work. Only some traces of fructose arising from isomerization of glucose have been identified. However, a total organic content (TOC) analysis has been carried out for each catalytic test and we always accounted for 100 mol.% of engaged carbon. It means that no other gaseous products are being formed.

**Table 2:** Conversion of cellobiose and selectivity in glucose.

**Catalyst**	**Conversion (%)**	**Selectivity in glucose (%)**
Blank	24	57
LHT-OX	19	87
SO_3_H/LHT-OX	25	47
RGO	40	24
SO_3_H/RGO	84	95
SX+	31	82
SO_3_H/SX+	81	96
MWCNT	24	47
SO_3_H/MWCNT	81	95
CB	20	52
SO_3_H/CB	57	70
GNP-M15	23	64
SO_3_H/GNP-M15	40	80
GNP-C750	29	57
SO_3_H/GNP-C750	94	80

The catalysts that are displaying a poor acidity, such as the SO_3_H/LHT-OX sample, do not improve the conversion at all. We can assess that there is a minimum amount of acid necessary to switch on the catalysis for the acidic hydrolysis. As shown in Figure 5, below 120 mmol/100 g the conversion remains low. Beyond this threshold value, the catalysts show a good activity, as long as sulfonic functions are present.

The quantity of catalyst used during the test also influences the activity. Several amounts of catalysts have been tested in the case of the SO_3_H/SX+ material (Figure 6). When the amount of catalyst is too low (20 mg), the catalyst is not active and the conversion is the same as the blank. When we increase the amount of catalyst, the acidic hydrolysis is triggered and the conversion is higher. However, the selectivity is excellent in all cases (much higher than the blank), independently of the amount of catalyst. Preliminary tests have been conducted on cellulose fibers (Sigma, reference C6288, crystalline, and high puritys) and 20% conversion (based on cellulose weight loss at the end of test) was observed.

Recyclability tests have been carried out with SO_3_H/SX+ and are shown on Figure 7. A slight decrease of the conversion from 81 to 67% is observed after the second run but the selectivity remains very high (97%). After the third run the conversion decreases again to reach 50% but stays quite stable for the next three runs. However, the selectivity is still reaching almost 100%. The plateau reached after three runs shows a conversion far better than the blank. It is shown here that the sulfonic functions are very robust and remain on the carbon material despite the test conditions.

**Figure 8:**
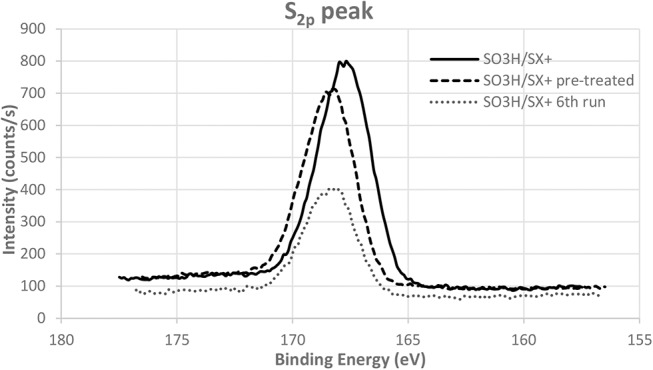
S_2p_ XPS peak for the functionalized SX+ before and after test and pre-treated catalyst.

The slight decrease of conversion can be incriminated to the removal of some unreacted sulfonic compounds that were just adsorbed on the carbon surface. Indeed the acidity of the catalyst after the sixth run is 129 mmol/100 g for 145 mmol/100 g before tests. Nevertheless, the selectivity into glucose stays high because of the presence of strong acid sulfonic functions, which are the active sites for this transformation, even after six runs. In order to confirm this effect, a pre-treatment in the same conditions as those of the catalytic tests but without cellobiose was performed on the catalyst. The acidity of the pre-treated catalyst is 116 mmol/100 g and match with the value obtained for the catalyst after six runs. Following the same trend than acidity, XPS analyses confirm that sulfonic functions are fewer but still present on the pre-treated catalyst and the catalyst after test (Figure 8). This demonstrates that the sulfonic groups are stable under the catalytic tests reaction conditions [130°C and N_2_ autogenic pressure (6 bar)]. It is also observed that the position of the sulfur peak is not shifted. This means that our sulfonic functions are not reduced during the catalytic test eluding this possibility as a deactivation phenomenon. Two other reasons for the slight drop in conversion observed in Figure 7 that might be cited are strong adsorption of reactants and/or products and coking. However, no significant weight increase of our catalyst has been observed after the test.

Moreover, the quantity of nitrogen in the used or pre-treated catalysts is very small compared to the quantity on the freshly synthesized catalyst. Reactants that were stacked on the carbon material are washed away under these conditions. The acidic functions that are still grafted on the solid are robust and that is why they can be used many times. The conversion and the selectivity obtained with the pre-treated catalyst, respectively 51 and 98%, are the same than the plateau reached after three runs. These results show that our sulfonic functions grafted with the diazonium coupling are much more stable than sulfonic functions generated by hot H_2_SO_4_ treatment (Wu et al., 2010; Zhong and Sels, 2018). Therefore, the main interest of these catalysts is their recyclability. We have therefore obtained highly active and stable acidic catalysts suitable for biomass hydrolysis reaction at low temperature and within a short reaction time.

## Discussion

Various (nano)carbon solids have been functionalized through a diazonium coupling reaction leading to strong acidic sulfonic functions grafted on their surface. Their presence has been confirmed by Boehm titration, XPS and TPD analyses. Hydrolysis of cellobiose into glucose under inert atmosphere in neutral water medium has been improved by all our functionalized catalysts, compared to a “blank” with no catalyst, or the unfunctionalized carbon materials. A maximum of 84% of conversion with 95% of selectivity toward glucose has been obtained with the SO_3_H/RGO catalyst. Moreover, these results have been obtained at a low temperature (130°C) in only 2 h which is promising for hydrolysis of more robust substrate like cellulose for example. It has been discovered that below a certain amount of acidic functions no catalytic improvement is observed. In our conditions, 120 mmol/100 g of catalyst acidity is the minimum amount to trigger the hydrolysis activity. It has also been shown that our catalysts are recyclable. Indeed the performance of the SO_3_H/SX+ catalyst after six runs is much better than the hydrolysis without any catalyst. Moreover, the observed slight decrease of activity has been assigned to some reactants stacked on the carbon material that are washed away, while the sulfonic functions well-bound on the surface remain on it after tests. It is demonstrating the robustness of these functions in comparison with other functionalization methods such as carbon materials treatment with sulfuric acid that would also weaken the material mechanical properties. These functionalized carbon can further be used as supports for metallic nanoparticles in order to prepare bifunctional catalysts. The acidic functions will hydrolyze cellobiose into glucose while the metallic center can transform the glucose into high value molecules like sorbitol or HMF.

## Data Availability Statement

All datasets generated for this study are included in the article/Supplementary Material.

## Author Contributions

SH is provider of funds and original ideas, supervised the experimental work, participated in data interpretation, and proof-read the manuscript. SC performed the experiments, collected and analyzed the data, and wrote the first draft of the manuscript.

## Conflict of Interest

The authors declare that the research was conducted in the absence of any commercial or financial relationships that could be construed as a potential conflict of interest.
