# Supplementary material for: Highly Efficient and Recyclable Catalysts for Cellobiose Hydrolysis: Systematic Comparison of Carbon Nanomaterials Functionalized With Benzyl Sulfonic Acids
Source: Front Chem. 2020 Apr 27;8:347. doi: 10.3389/fchem.2020.00347 (PMC7198230; doi:10.3389/fchem.2020.00347)
Supplement: Supplementary file 1 [file Data_Sheet_1.PDF]

Highly efficient and recyclable catalysts for cellobiose hydrolysis: systematic comparison of carbon nanomaterials functionalized with benzyl sulfonic acids

Samuel Carlier, Sophie Hermans*

Université catholique de Louvain, IMCN Institute, Place L. Pasteur 1, 1348 Louvain-la-Neuve, Belgium

** Corresponding author: e-mail: sophie.hermans@uclouvain.be*

Supporting information

S1. XPS spectra of S2p for all carbon materials before and after functionalization

S2. XPS analyses for N1s and C1s for representative carbon samples

S3. Relationship between BET surface area and acidity increase/S amount increase.

S4. Comparison with literature results in term of catalyst acidity

S5. TPD analyses

S6. SO_2 signal followed by MS during TPD analysis

S7. Hydrolysis mechanism

S1. XPS spectra of S_{2p} for all carbon materials before and after functionalization

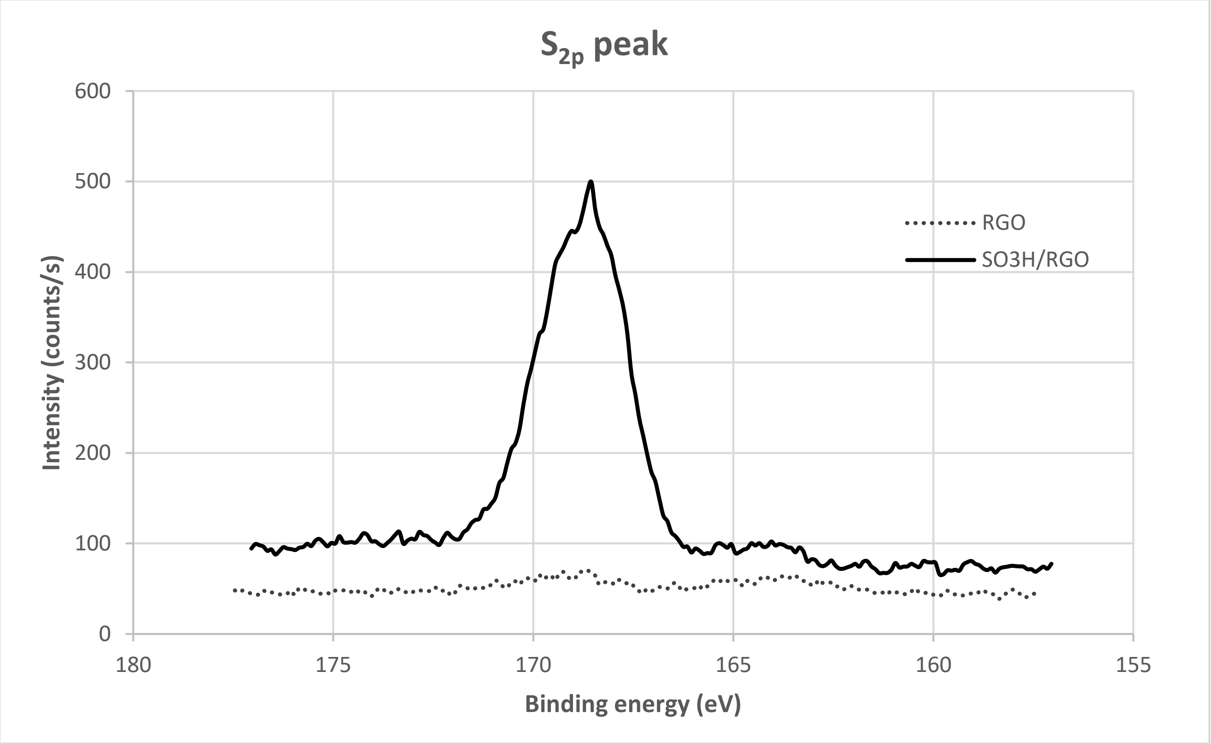

Figure 1: S_{2p} XPS peak for RGO and functionalized RGO samples.

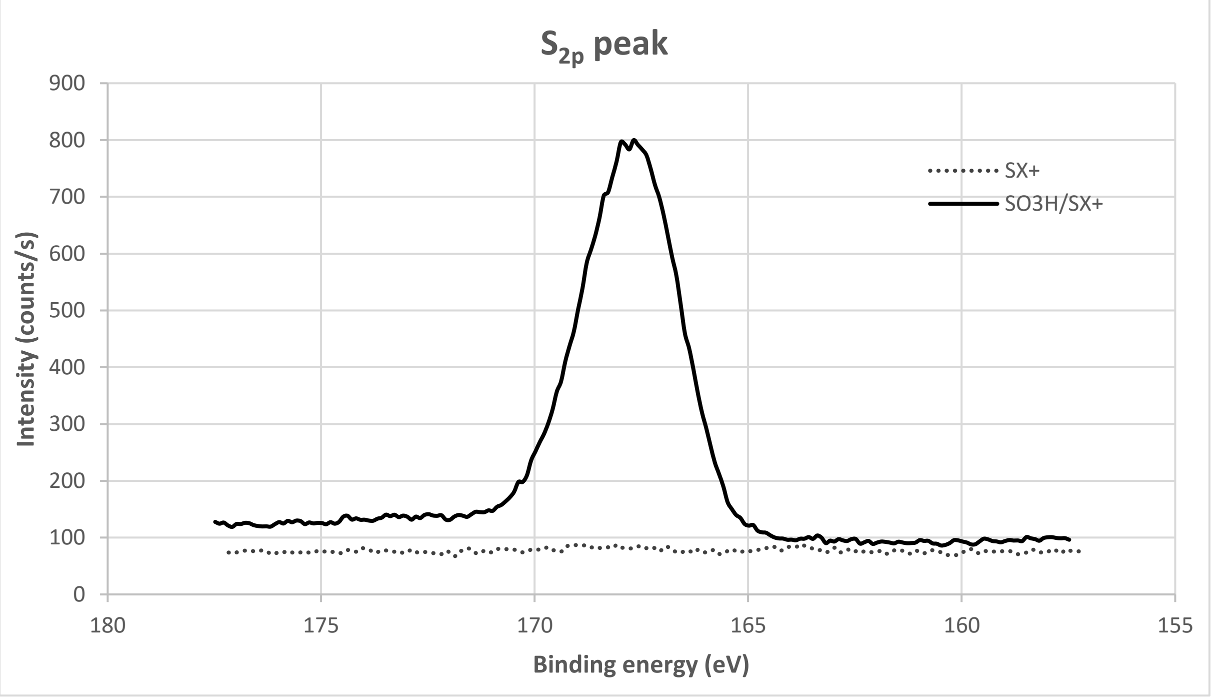

Figure 2: S_{2p} XPS peak for SX+ and functionalized SX+ samples.

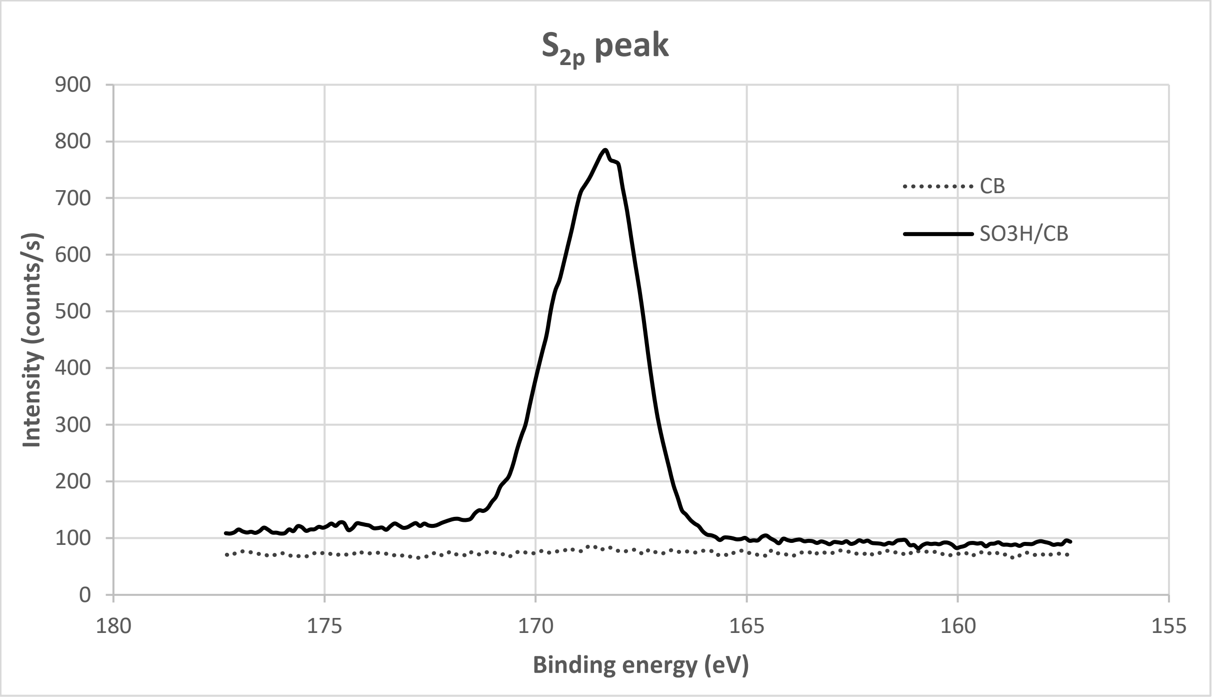

Figure 3: S_{2p} XPS peak for CB and functionalized CB samples.

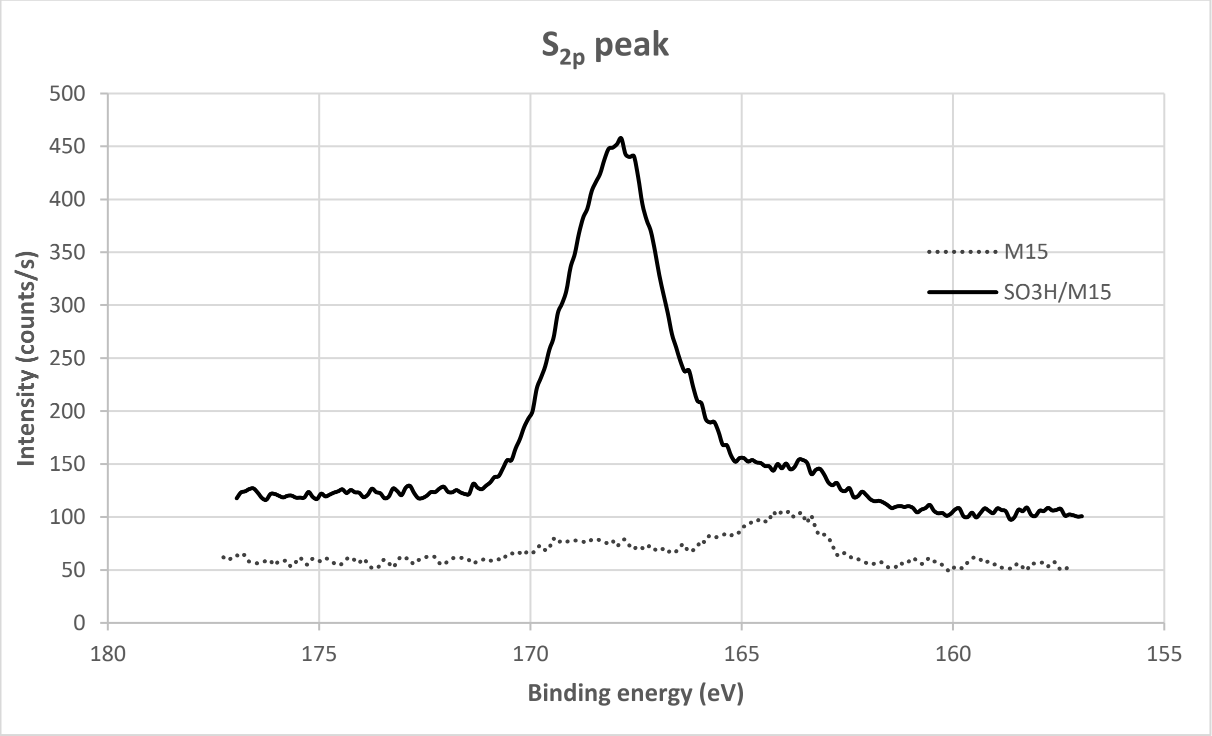

Figure 4: S_{2p} XPS peak for M15 and functionalized M15 samples.

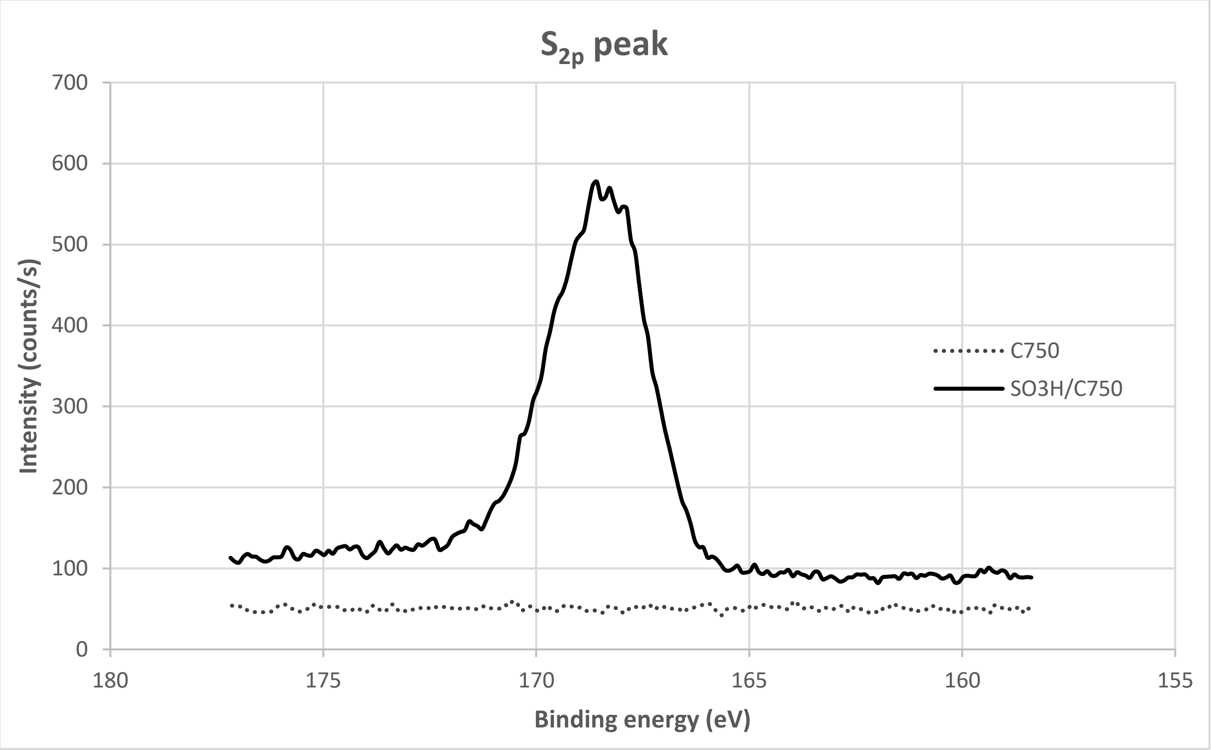

Figure 5: S_{2p} XPS peak for C750 and functionalized C750 samples.

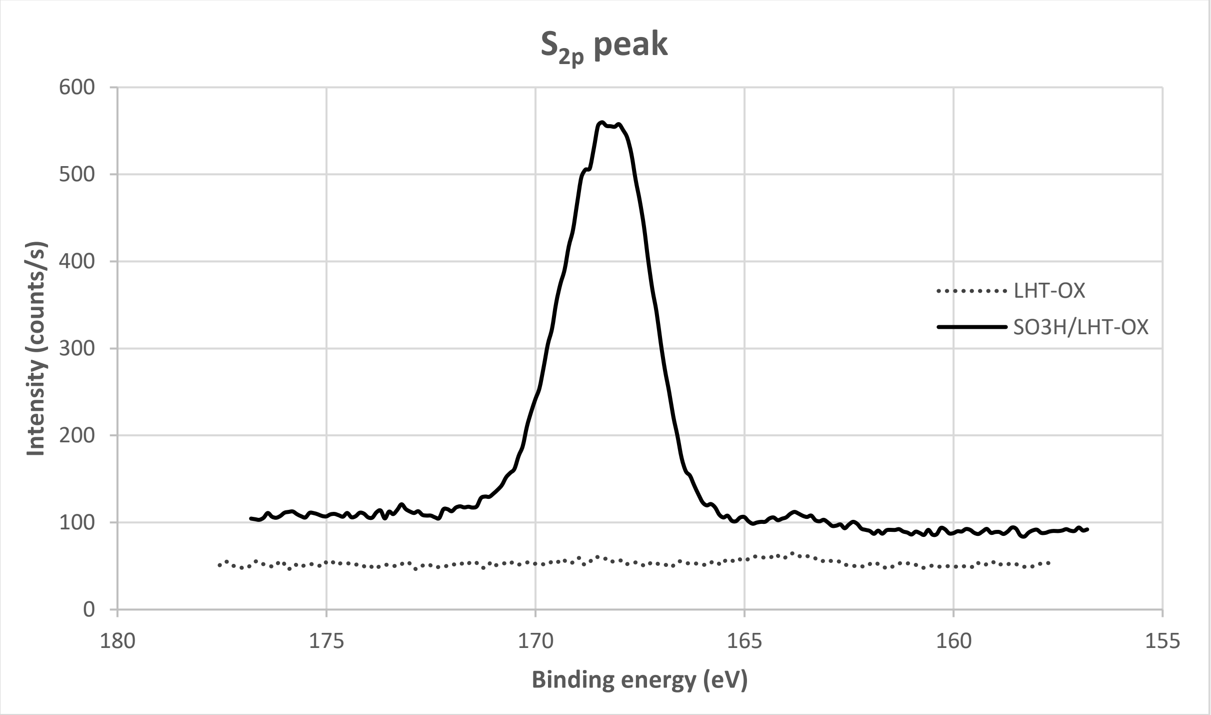

Figure 6: S_{2p} XPS peak for LHT-OX and functionalized LHT-OX samples.

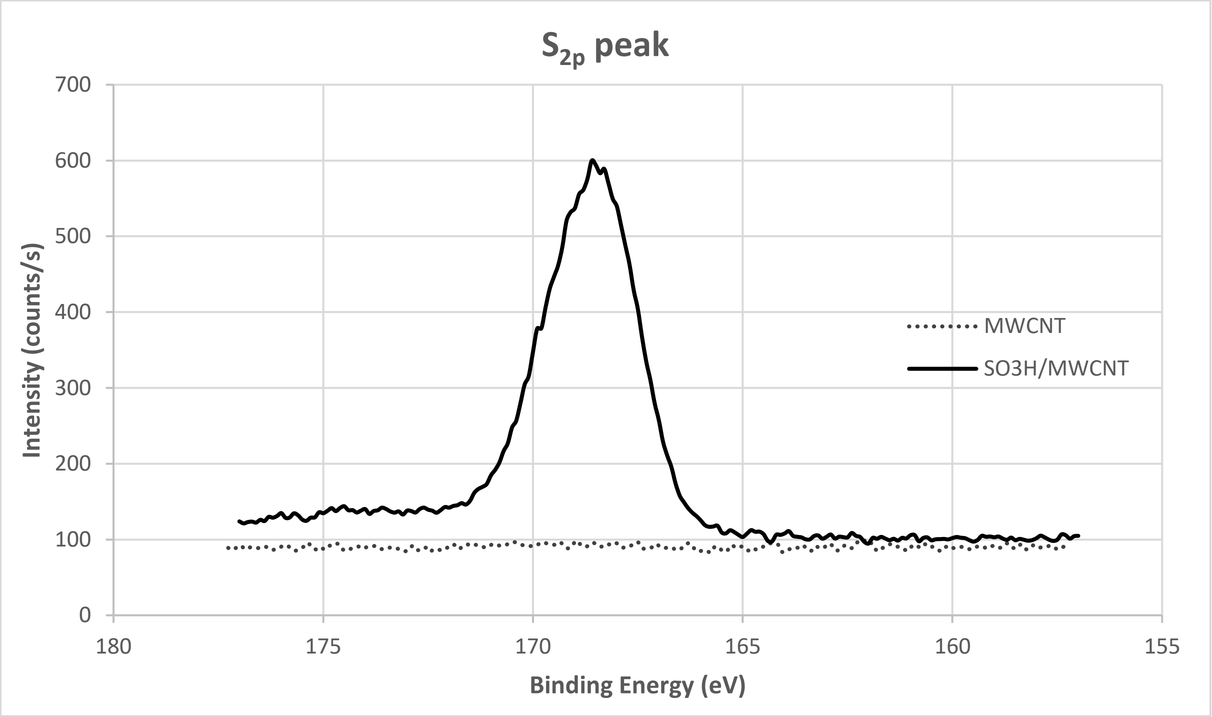

Figure 7: S_{2p} XPS peak for MWCNT and functionalized MWCNT samples.

S2. XPS analyses for N_{1s} and C_{1s} for representative carbon samples

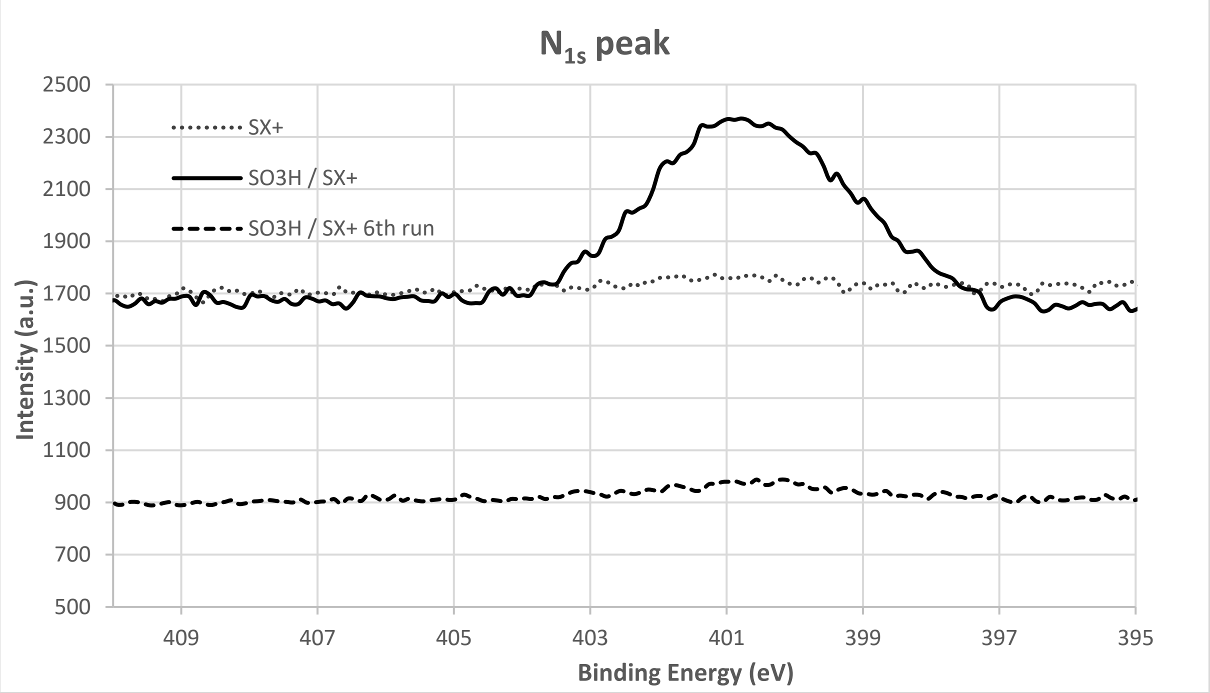

Figure 8: N_{1s} peak by XPS for pristine and functionalized SX+ before and after catalytic test

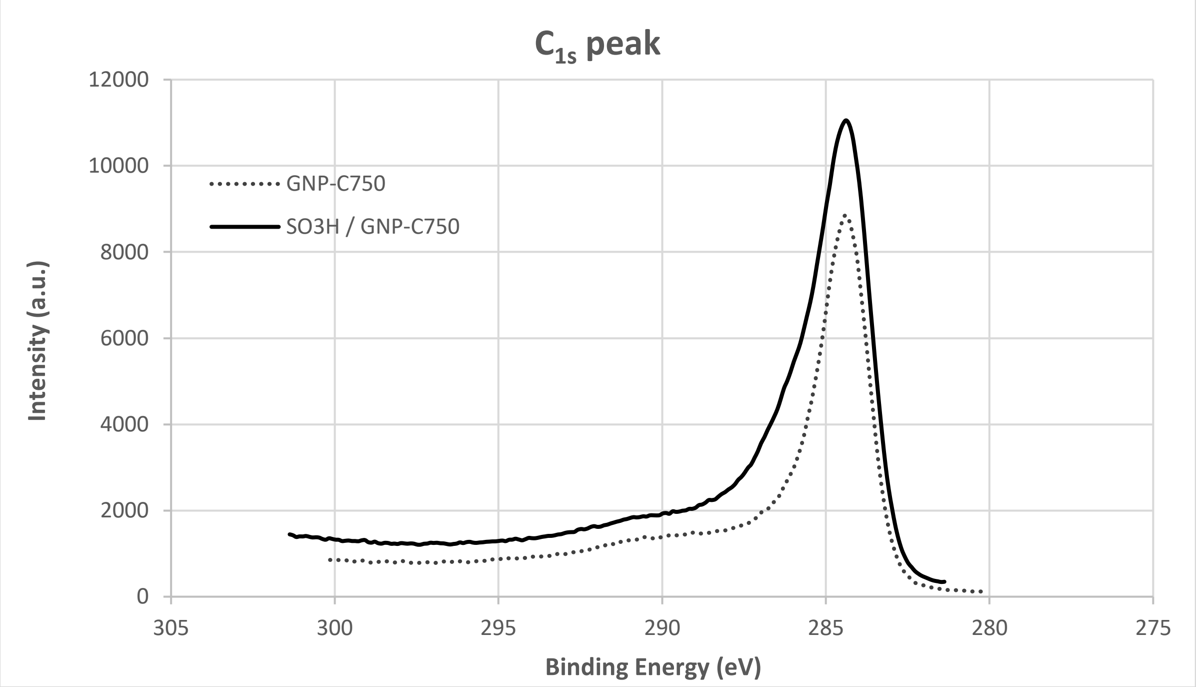

Figure 9: C_{1s} peak by XPS for pristine and functionalized GNP-C750

S3. Relationship between BET surface area and acidity increase/S amount increase.

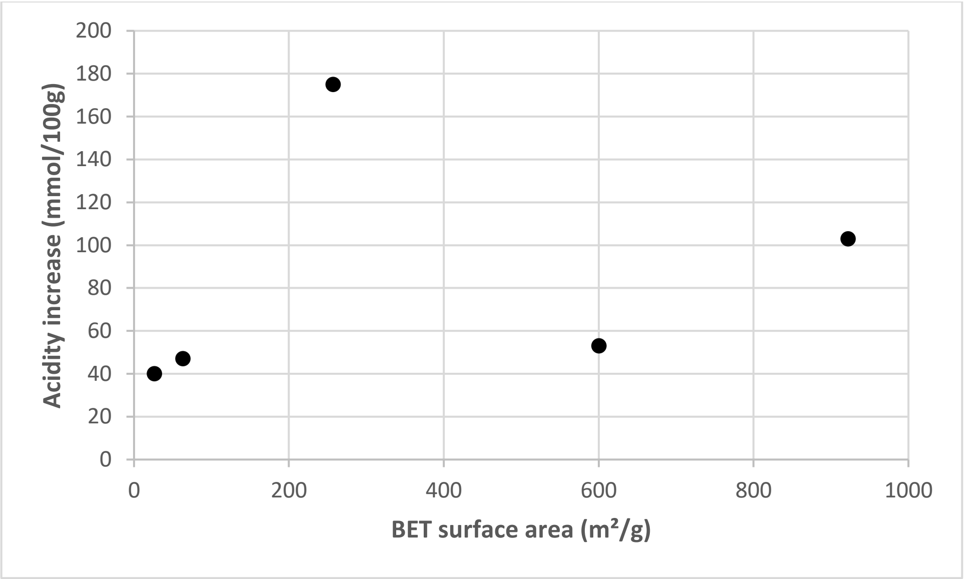

Figure 10: Acidity increase as a function of BET surface area

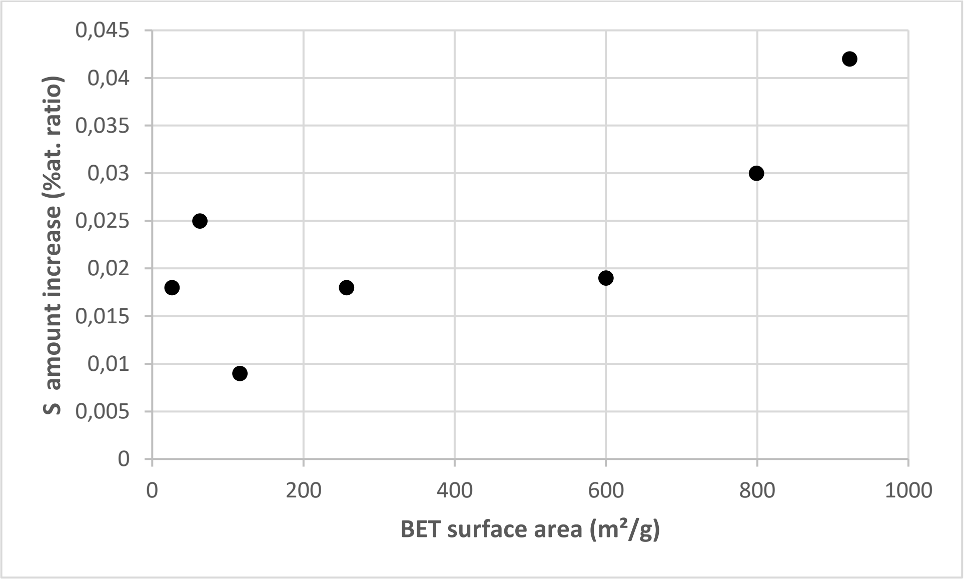

Figure 11: S amount increase measured by XPS as a function of BET surface area

S4. Comparison with literature results in term of catalyst acidity

Sample	Acidity (mmol/100 g)
SO_3H/MWCNT (our catalyst)	189
SO_3H/LHT-OX (our catalyst)	50
Sulfonated carbon nanofibers (Stellwagen et al., 2013)	63
Sulfonated activated carbon (Huang and Fu, 2013)	190
BC-SO_3H (Huang and Fu, 2013)	198
SC-SO_3H (Huang and Fu, 2013)	215
CMK-3-SO_3H (Huang and Fu, 2013)	239
Cellulose based sulfonated cellulose based amorphous carbon (Stellwagen et al., 2013)	108
HY zeolite (Huang and Fu, 2013)	136
Aluminosilicates (H-beta) (Onda et al., 2008)	105
$HNbMoO_6$ (Huang and Fu, 2013)	190
Amberlyst-15 (Onda et al., 2008)	470

References

Huang, Y. B., and Fu, Y. (2013). Hydrolysis of cellulose to glucose by solid acid catalysts. *Green Chem.* 15, 1095–1111. doi:10.1039/c3gc40136g.

Huber, G. W., Iborra, S., and Corma, A. (2006). Synthesis of transportation fuels from biomass: Chemistry, catalysts, and engineering. *Chem. Rev.* 106, 4044–4098. doi:10.1021/cr068360d.

Onda, A., Ochi, T., and Yanagisawa, K. (2008). Selective hydrolysis of cellulose into glucose over solid acid catalysts. *Green Chem.* 10, 1033–1037. doi:10.1039/b808471h.

Stellwagen, D. R., Van Der Klis, F., Van Es, D. S., De Jong, K. P., and Bitter, J. H. (2013). Functionalized carbon nanofibers as solid-acid catalysts for transesterification. *ChemSusChem* 6, 1668–1672. doi:10.1002/cssc.201300372.

S5. TPD analyses

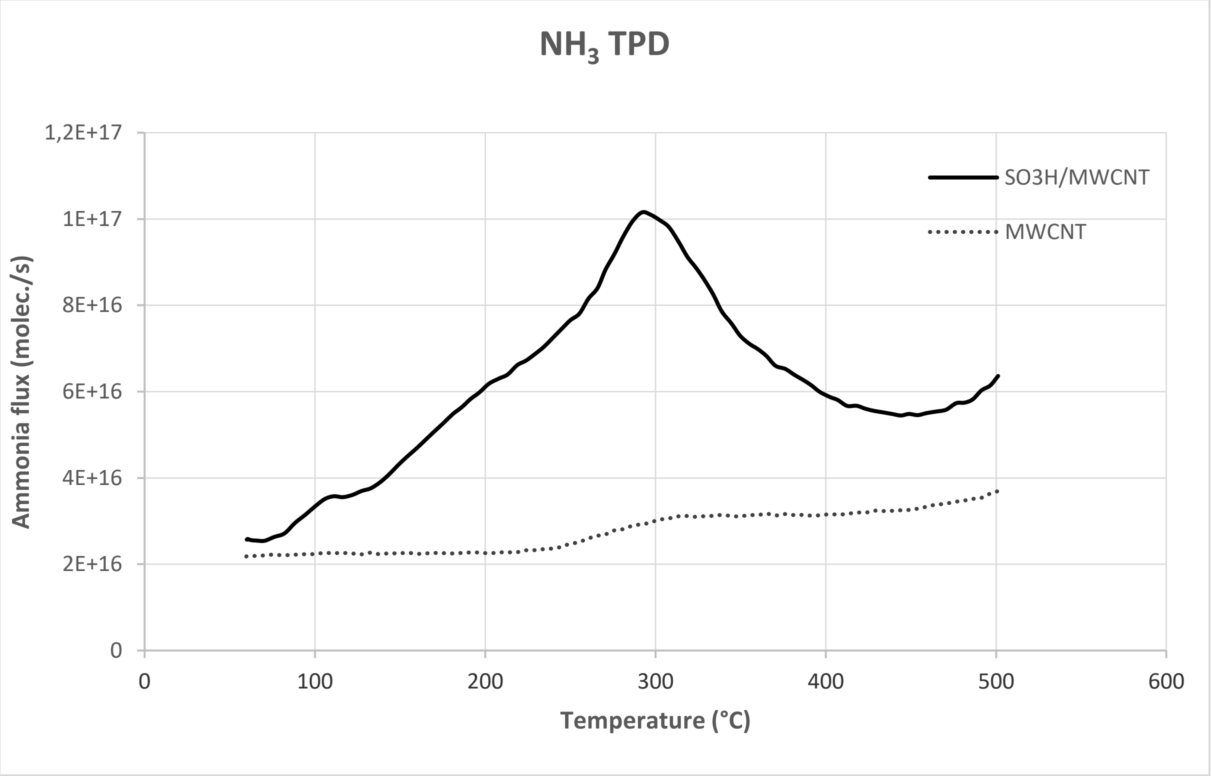

Figure 12: NH_3 flux as a function of temperature for MWCNT and functionalized MWCNT

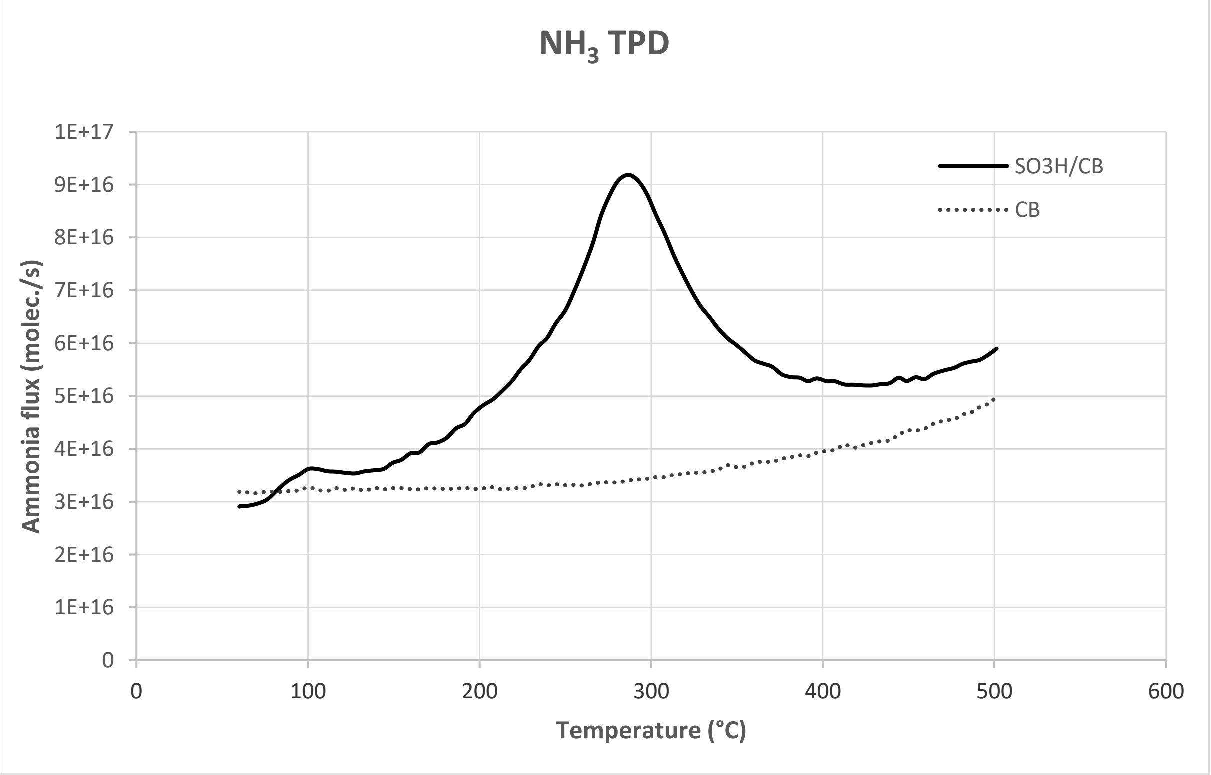

Figure 13: NH_3 flux as a function of temperature for CB and functionalized CB

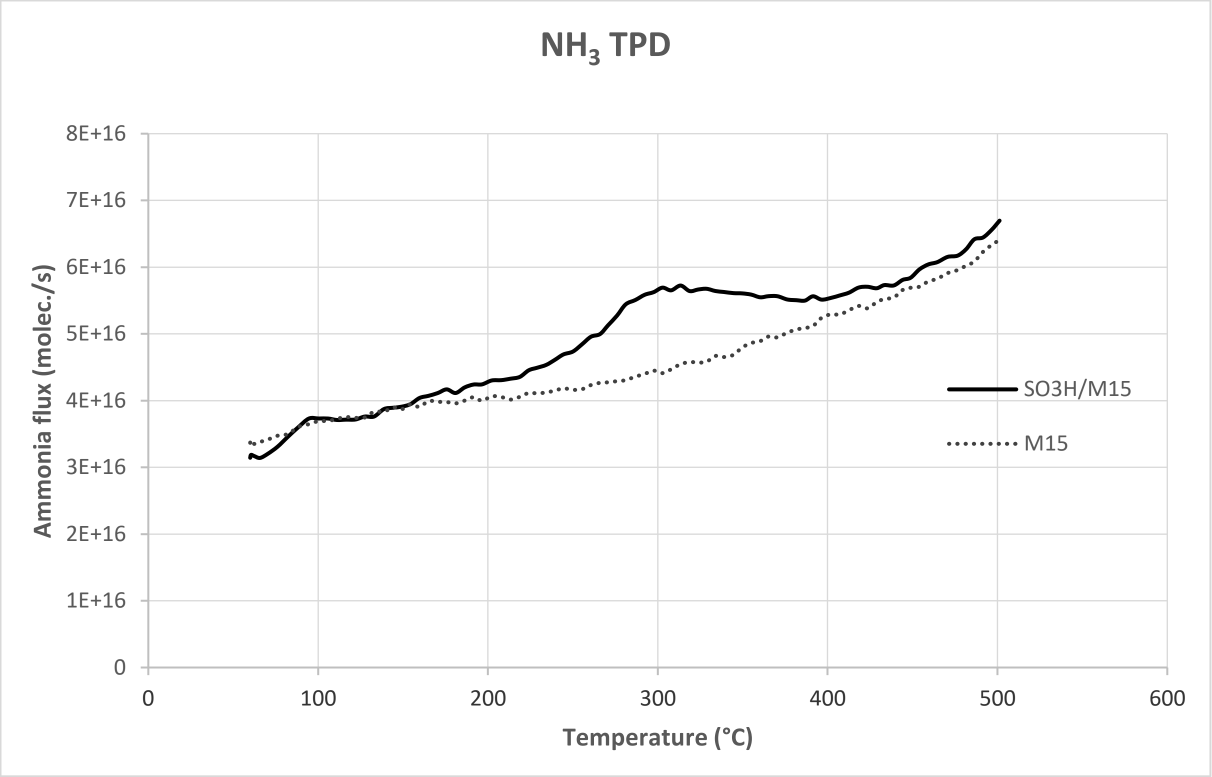

Figure 14: NH_3 flux as a function of temperature for M15 and functionalized M15

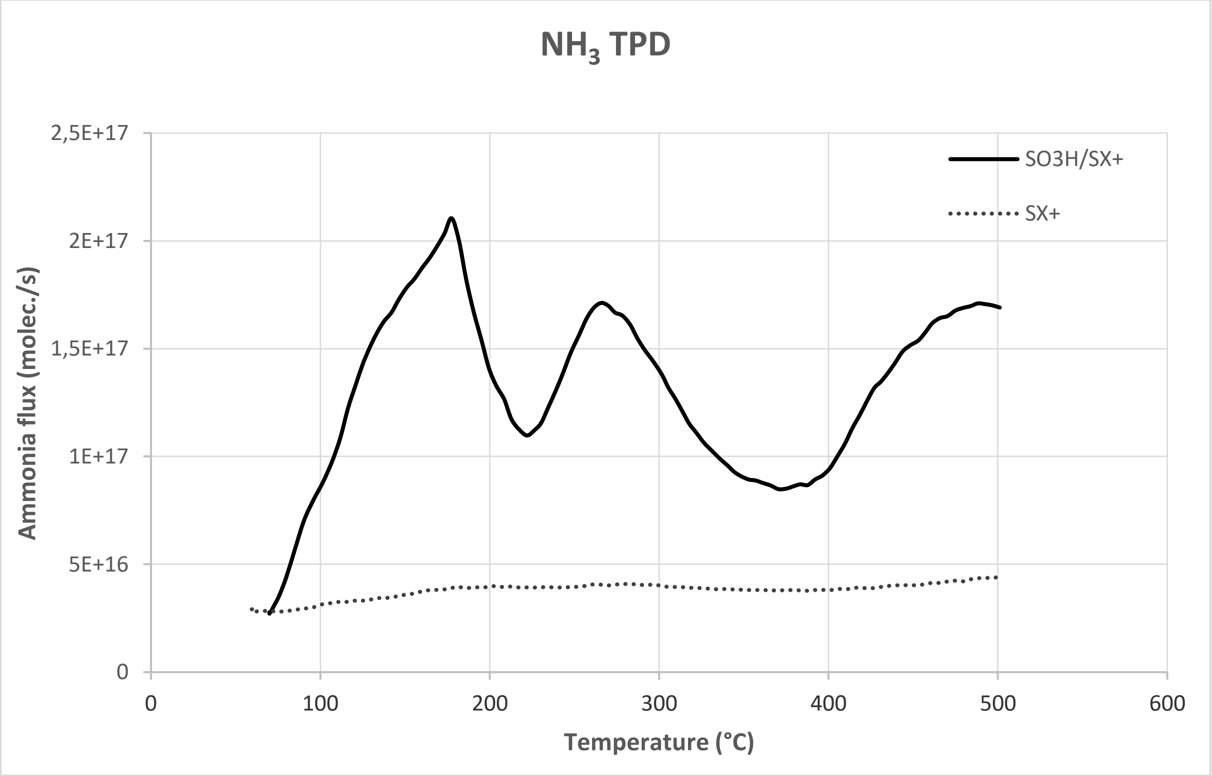

Figure 15: NH_3 flux as a function of temperature for SX+ and functionalized SX+

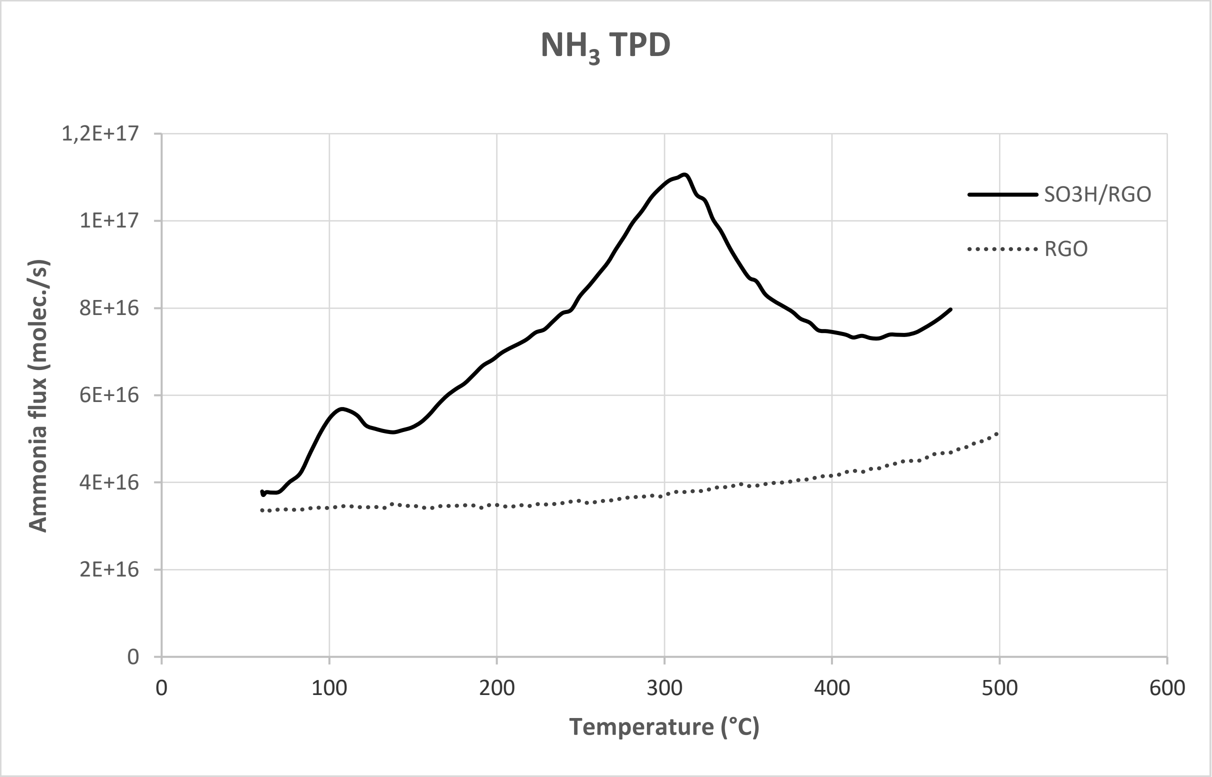

Figure 16: NH_3 flux as a function of temperature for RGO and functionalized RGO

S6. SO_2 signal followed by MS during TPD analysis

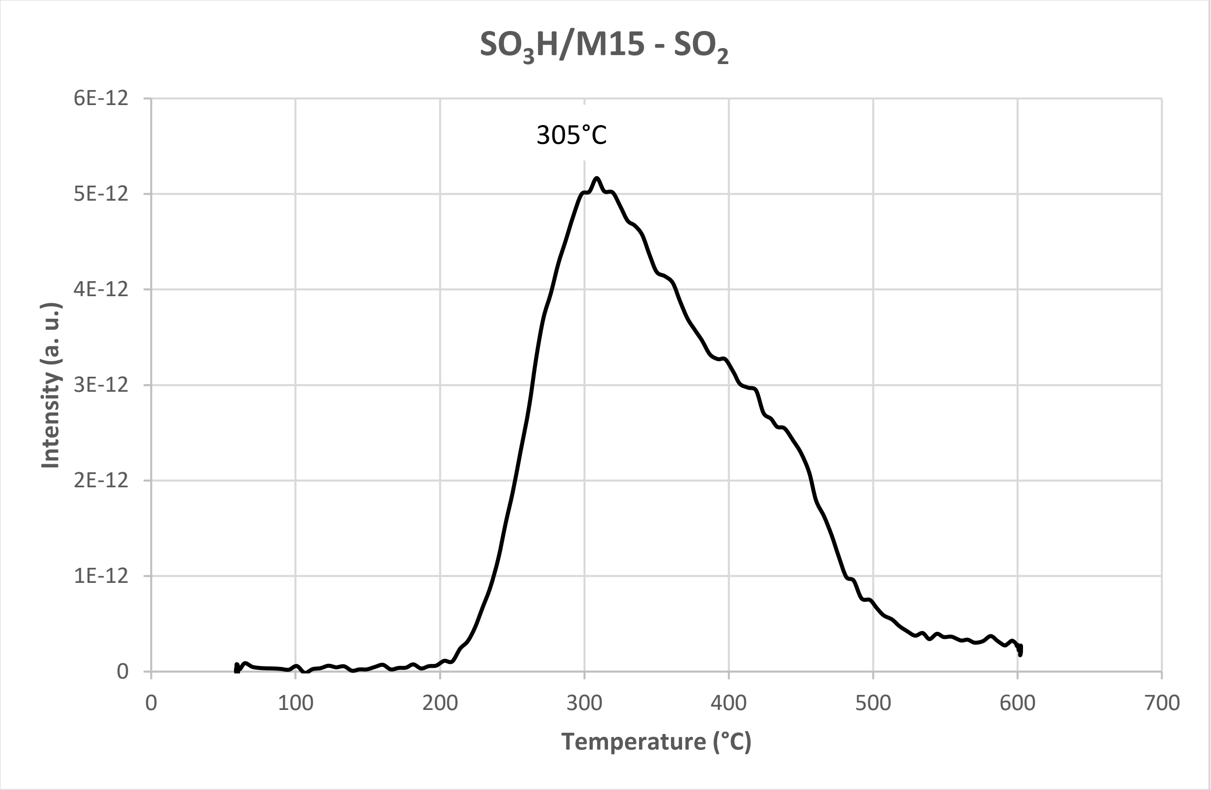

Figure 17: SO_2 signal followed by MS during the TPD analysis of functionalized M15

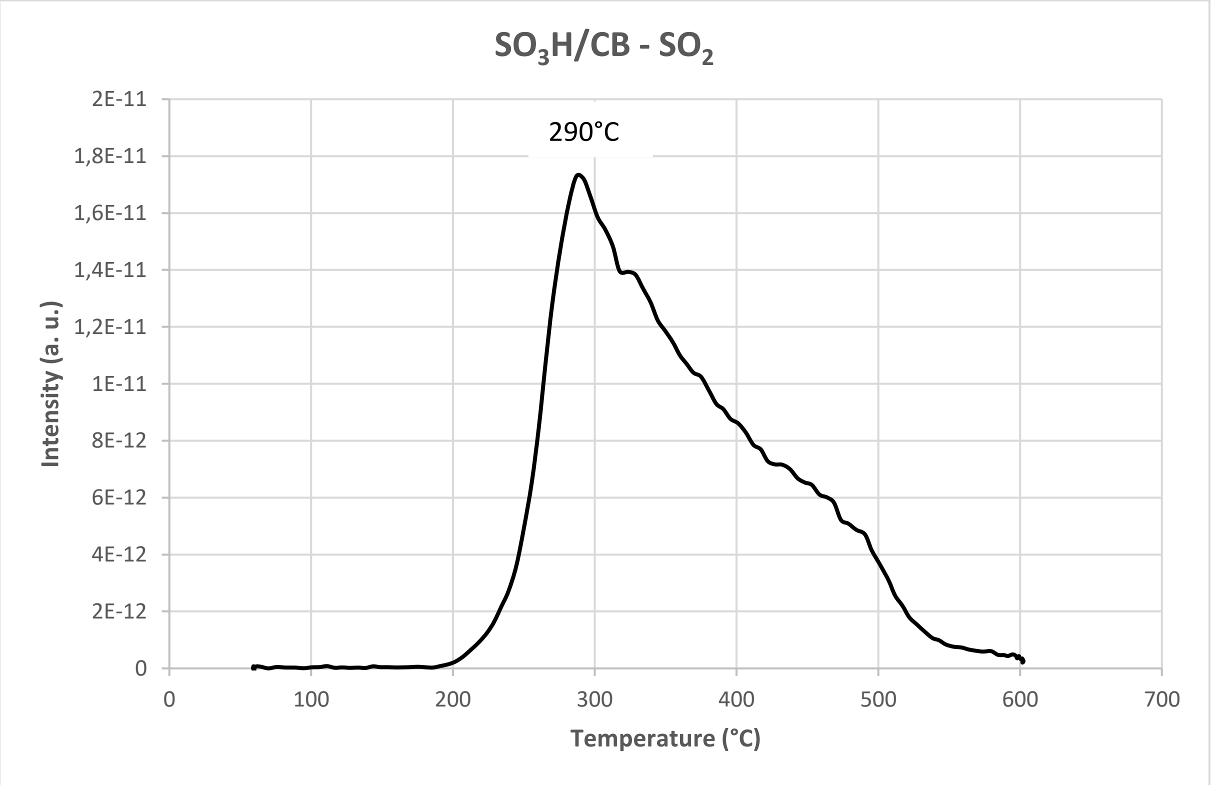

Figure 18: SO_2 signal followed by MS during the TPD analysis of functionalized CB

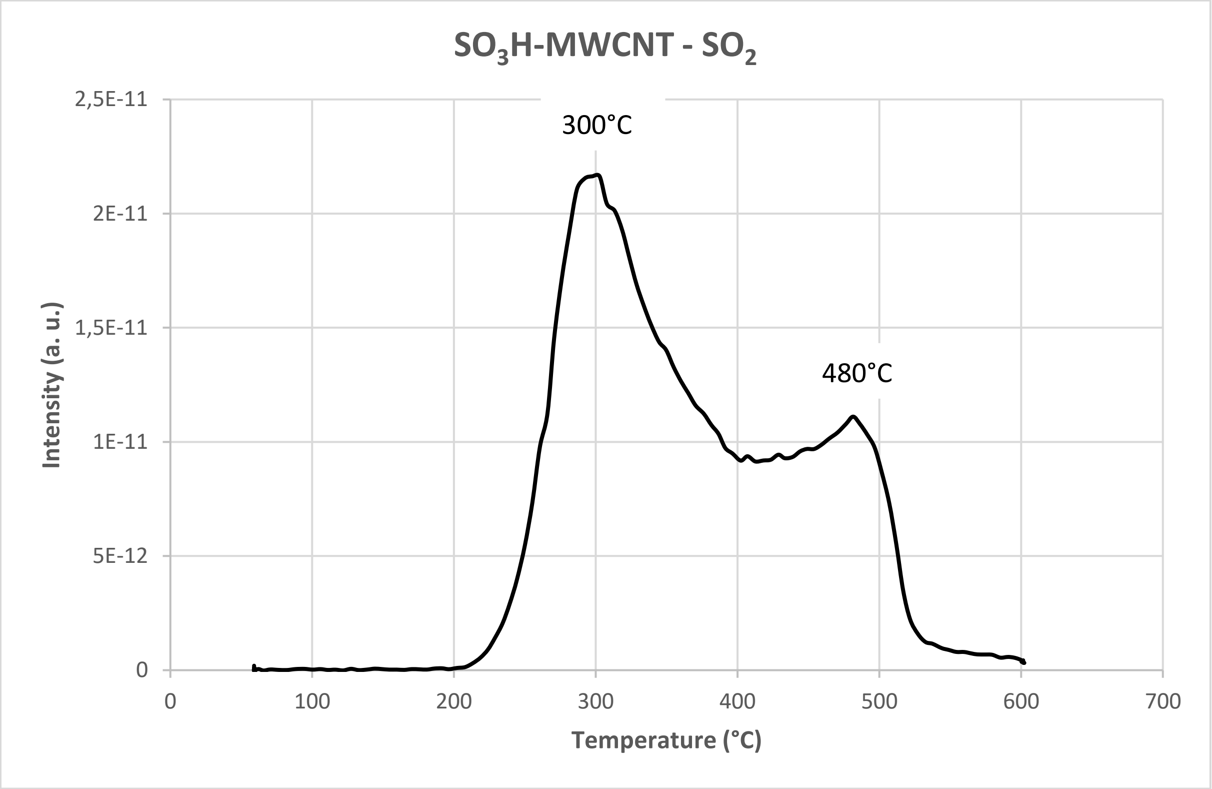

Figure 19: SO_2 signal followed by MS during the TPD analysis of functionalized MWCNT

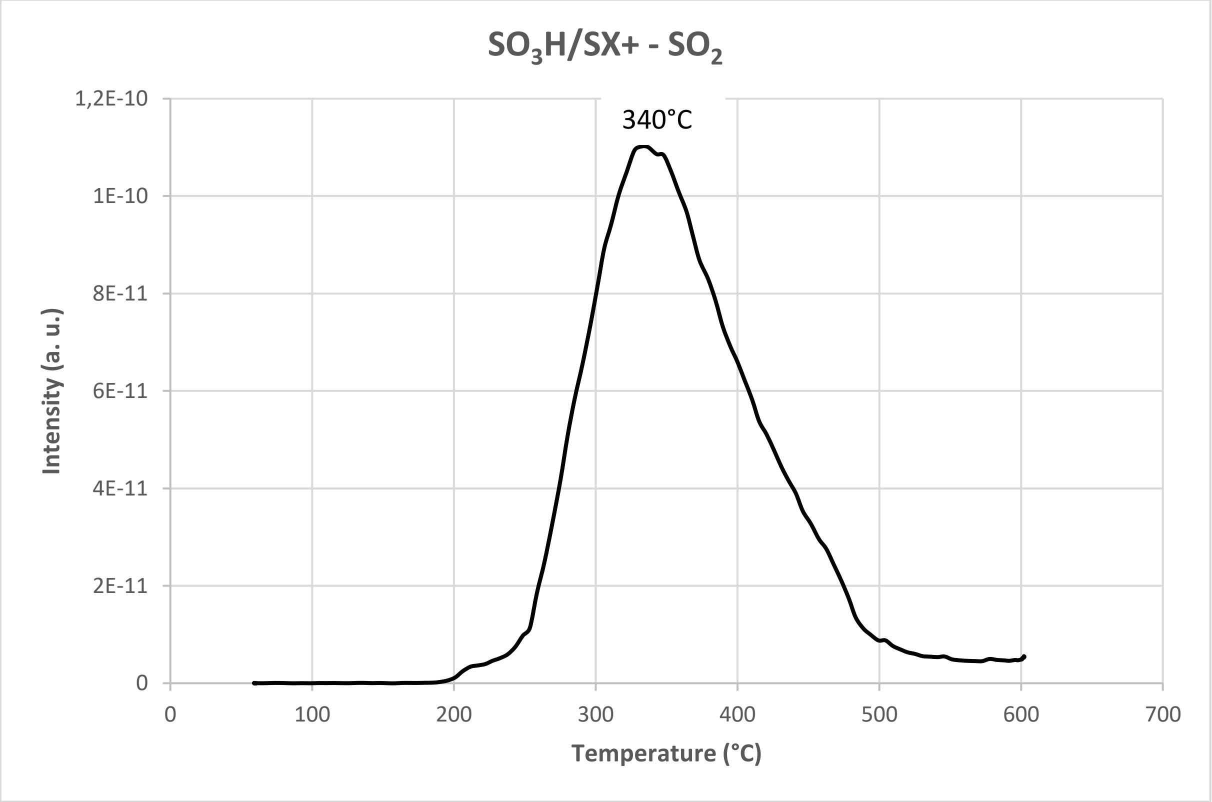

Figure 20: SO_2 signal followed by MS during the TPD analysis of functionalized SX+

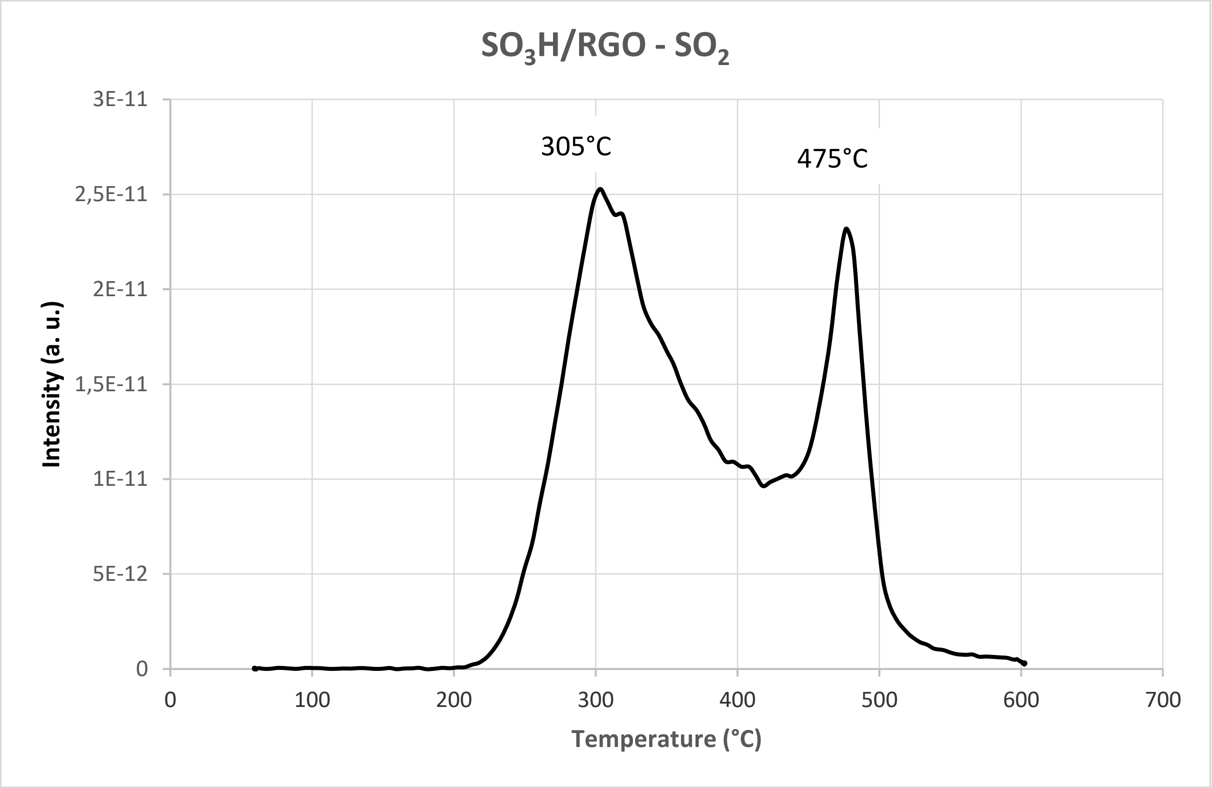

Figure 21: SO_2 signal followed by MS during the TPD analysis of functionalized RGO

S7. Hydrolysis mechanism

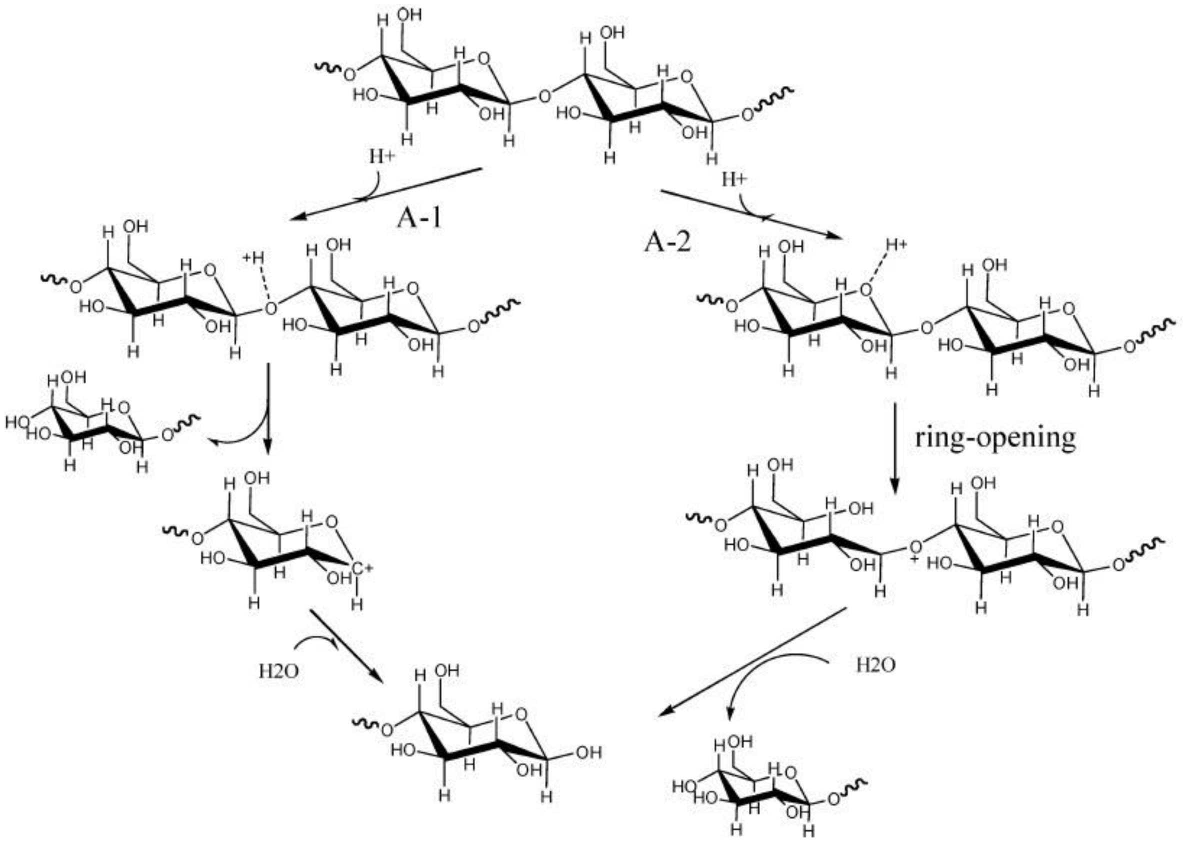

Figure 22 : Hydrolysis mechanism giving glucose from cellulose by acidic catalysis, from (Huber et al., 2006)
